# Acute ischemic stroke with contralateral convexal subarachnoid hemorrhage: two cases report

**DOI:** 10.1186/s12883-019-1364-9

**Published:** 2019-06-18

**Authors:** Yayun Cao, Jie Cao, Suqiong Ji, Shabei Xu, Chenchen Liu

**Affiliations:** Department of Neurology, Tongji Hospital, Tongji Medical College, Huazhong University of Science and Technology, 1095 Jiefang Avenue, Wuhan, Hubei 430030 People’s Republic of China

**Keywords:** Convexal subarachnoid hemorrhage, Acute ischemic stroke, Internal carotid artery occlusion, Cardioembolic stroke, Case report

## Abstract

**Background:**

Convexal subarachnoid hemorrhage (cSAH) is characterized by isolated bleeding in one or a few adjacent sulci and has diverse etiologies and symptoms. Acute ischemic stroke co-occurring with cSAH has been infrequently reported. Nearly all cases of cSAH have been described to occur on the side with acute ischemic stroke, and it is unusual for cSAH to occur on the opposite side of the infarct territory.

**Case presentation:**

Our report presents two cases of acute ischemic stroke associated with contralateral cSAH. The first patient had left atherosclerotic internal carotid artery (ICA) occlusion with developing right parietal cSAH. The other patient developed left parietal cSAH in the setting of right ICA occlusion caused by cardiogenic embolism with acute right cerebral hemisphere infarction. Both patients remained clinically stable with good prognosis after antithrombotic treatment.

**Conclusions:**

Our report suggest that cSAH may simultaneously occur on the opposite side of an infarction. Although there is no consensus on the etiology and treatment of this rare phenomenon, cSAH did not lead to a poor outcome in our patients.

## Background

Nontraumatic convexal subarachnoid hemorrhage (cSAH) is a rare presentation of subarachnoid hemorrhage (SAH) localized to one or few sulci of the brain [1]. Various vascular and nonvascular causes of cSAH have been proposed [2]. According to previous studies, internal carotid artery (ICA) stenosis or occlusion is a common etiology of cSAH, and nearly all cSAH cases in those studies were reported to be associated with ipsilateral acute ischemic stroke [3–9]. To our knowledge, only one case of embolic stroke associated with contralateral cSAH has been reported [10]. We report two cases of acute ischemic stroke with contralateral cSAH.

## Case presentation

### Case 1

**Fig. 1:**
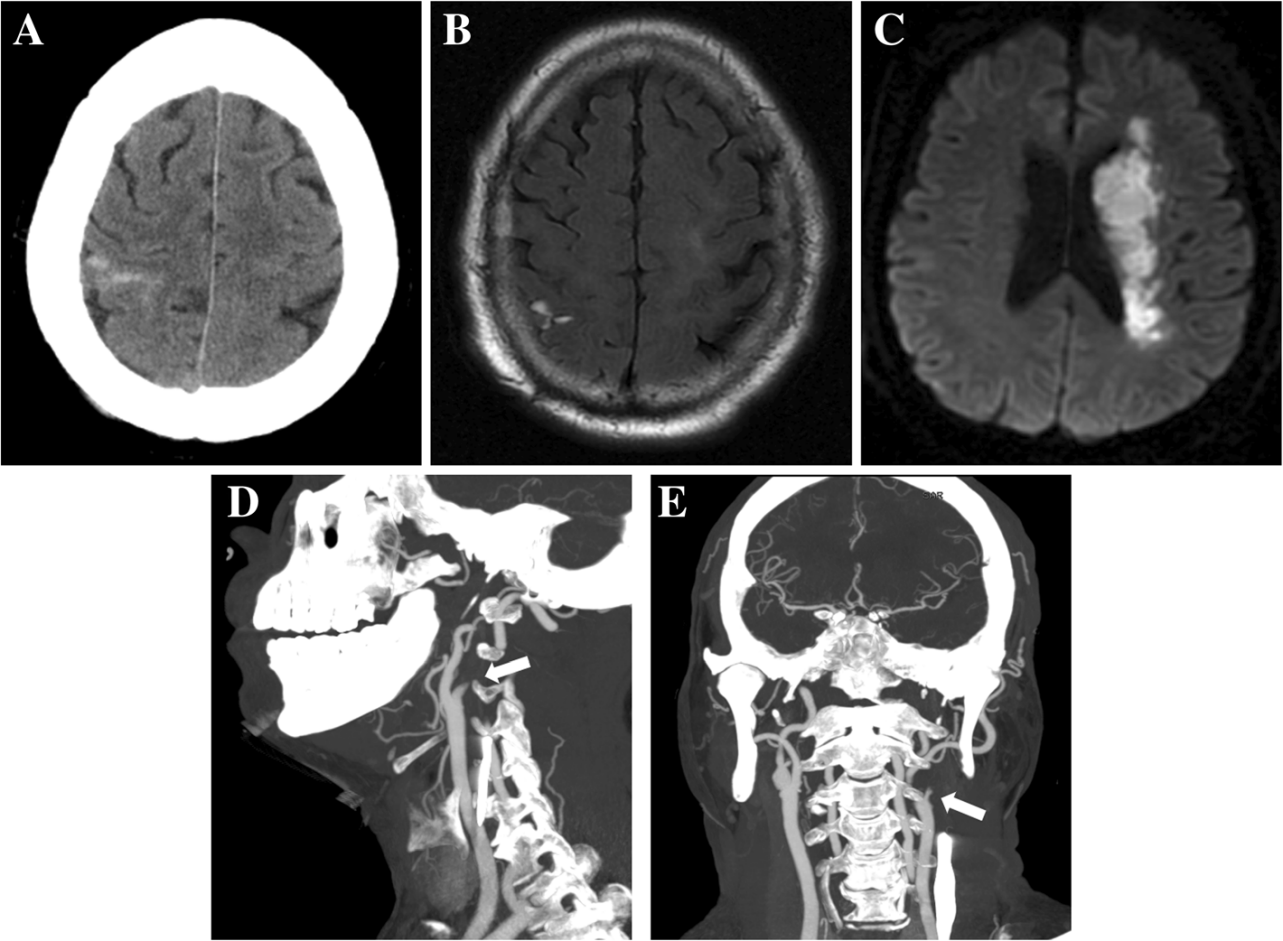
A 68-year-old male patient with left ICA occlusion and acute ischemic stroke with contralateral cSAH. **a** CT showed SAH on the right parietal convexity. **b** T2-weighted fluid-attenuated inversion recovery MRI confirmed a right parietal SAH. **c** Diffusion-weighted imaging showed high intensity in the region of the left middle cerebral artery. CT-angiography demonstrated occlusion of the left ICA (arrow) **d** and compensatory flow from the right ICA via the anterior communicating artery **e**

A 68-year-old man with a history of smoking and hypertension was admitted to our hospital for right-sided weakness and aphasia. On admission, his blood pressure was 170/108 mmHg. Neurologic examination showed right hemiplegia, facial paralysis and aphasia. Brain computed tomography (CT) showed a right parietal cSAH (Fig. 1a). Moreover, results from magnetic resonance imaging (MRI) T2-weighted fluid-attenuated inversion recovery were compatible with cSAH, which was localized to the sulcus in the right parietal lobe (Fig. 1b), Diffusion-weighted imaging was performed and showed hyperintense lesions in the distribution of the left middle cerebral artery (Fig. 1c). Further evaluation with CT-angiography showed occlusion of the left ICA and compensatory flow from the right ICA via the anterior communicating artery (Fig. 1d and e). Laboratory tests, including evaluations for inflammation, coagulation parameters, autoantibodies and neoplastic markers, were all unremarkable. Cerebral amyloid angiopathy (CAA) was excluded because of the absence of microbleeds on susceptibility-weighted imaging. There was no evidence of posterior reversible encephalopathy syndrome, without typical parieto-occipital vasogenic edema on MRI. Moreover, color Doppler ultrasonography showed *atherosclerotic plaque* formation *in the* bilateral *carotids and lower extremities.* Thus, the diagnosis of large artery atherosclerosis stroke was confirmed and full anti-atherosclerosis therapy was initiated (aspirin and atorvastatin). At 3 months’ follow-up, the patient had residual right-side limbs weakness and mild disability (modified Rankin Scale 2).

### Case 2

**Fig. 2:**
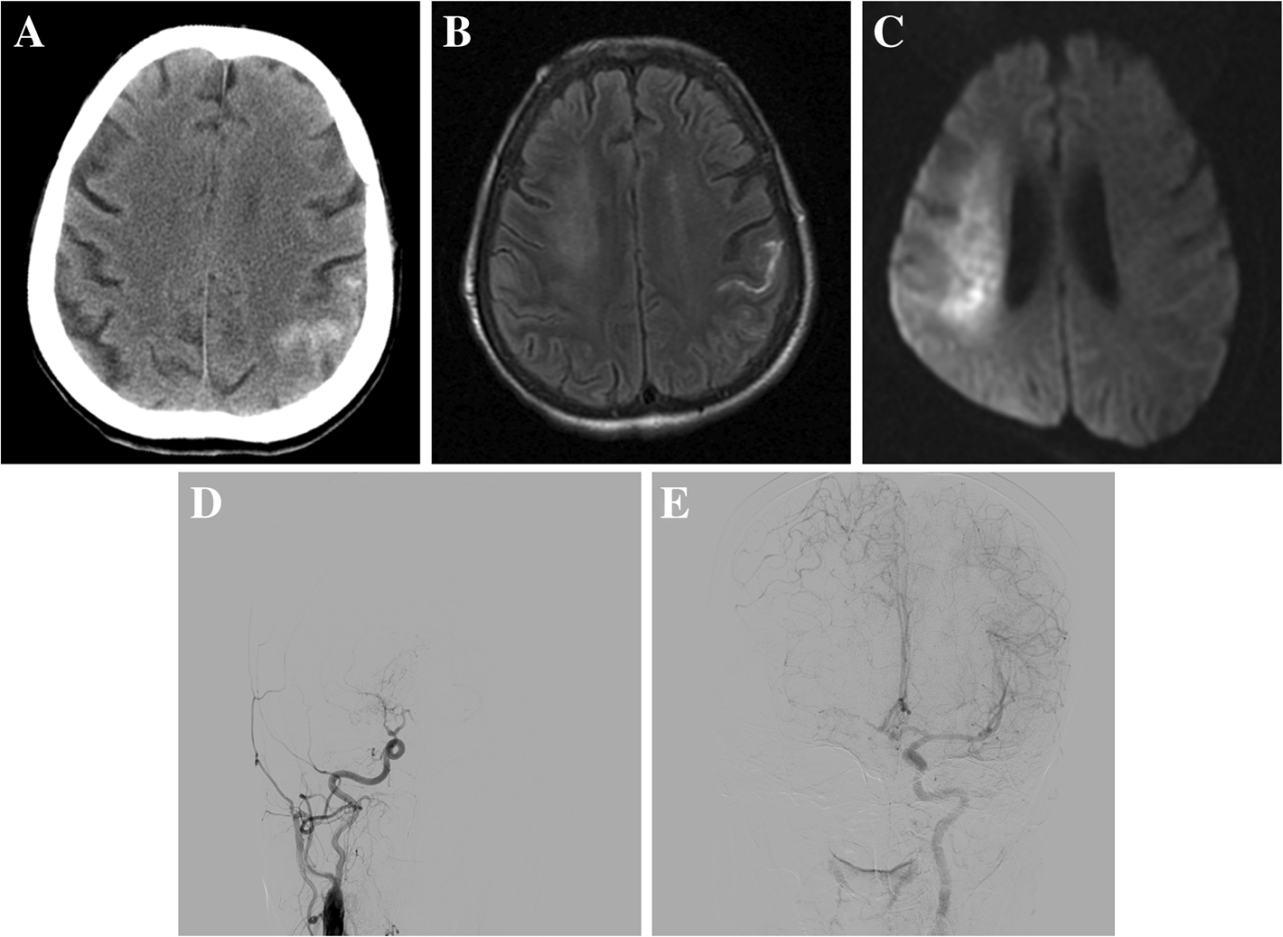
A 56-year-old female patient with cSAH due to cardioembolic stroke. **a** CT demonstrated a left parietal SAH. **b** T2-weighted fluid-attenuated inversion recovery MRI showed high-signal intensity on the left parietal convexity. **c** Diffusion-weighted imaging demonstrated acute ischemia in the right middle cerebral artery region. Digital subtraction angiography showed occlusion of the right ICA (**d**), and compensatory flow from the ipsilateral anterior cerebral artery via the leptomeningeal artery (**e**)

A 56-year-old woman with a history of untreated rheumatic heart disease for 20 years developed left-sided weakness associated with headache for 2 days. There was no history of hypertension, diabetes or hyperlipidemia. Her blood pressure at presentation was 91/58 mmHg. On neurological examination, she was somnolent with a binocular gaze to the right side. Her left limb muscle strength was grade 0/5 with hyperreflexia and positive pathological signs after hospitalization. CT revealed a left parietal cSAH (Fig. 2a). MRI with diffusion-weighted imaging confirmed the diagnosis of acute right cerebral hemisphere infarction and left parietal cSAH (Fig. 2b and c), without signs of microbleeds on susceptibility-weighted imaging. Digital subtraction angiography performed the next day showed right ICA occlusion (Fig. 2d and e). Laboratory findings revealed no evidence of vasculitis, infections, and coagulation disorders. Transthoracic echocardiography showed rheumatic heart disease with severe aortic stenosis and decreased left ventricular diastolic function. The ischemia was classified as ICA occlusion due to cardioembolic stroke. Warfarin was initiated after 2 weeks. Three months later, she underwent elective aortic valve replacement and continued long-term warfarin therapy. She could self-care after a follow-up period of 6 months and had modified Rankin Scale of 2.

## Discussion and conclusions

Nontraumatic SAH is mainly caused by aneurysm rupture, but not all cases have an aneurysmal origin [11]. cSAH is a rare and heterogeneous entity, and is characterized by bleeding along the cortical convexity. The prevalence of cSAH is reported to be 5–18% of all spontaneous SAH [1, 7, 12–14]. Diverse causes of cSAH have been described including CAA, reversible cerebral vasoconstriction syndrome (RCVS), posterior reversible encephalopathy syndrome, ICA stenosis or occlusion, cerebral venous thrombosis, drug misuse, vasculitis, vascular malformation, brain tumors and abscesses [1, 2, 12]. cSAH may occur simultaneously with acute ischemic stroke, but this co-occurrence is particularly rare. There are various reasons for this association, such as ICA stenosis or occlusion, artery dissection, RCVS, CAA, and cardioembolism [3–9, 15]. However, the most common cause of cSAH with acute ischemic stroke is ICA stenosis or occlusion [7]. Moreover, cSAH has been described to occur invariably on the side with acute ischemic stroke, and it is notably unusual for cSAH to occur on the opposite side of the infarct territory. To date, there have been few reports about infarction with contralateral cSAH caused by RCVS, ICA dissection, embolic stroke of undetermined source, and CAA [8–10, 15]. This paper presented two cases of acute ischemic stroke with contralateral cSAH due to ICA atherosclerotic occlusion and cardioembolic stroke, respectively.

The pathophysiology of the association between acute ischemic stroke and cSAH is not fully understood. The current leading theory suggests that cSAH may occur in the setting of severe atherosclerotic stenosis or large artery occlusion, potentially by a mechanism similar to hemorrhage caused by moyamoya disease [2, 16], the acute alteration in hemodynamics may lead to cSAH. Plaque rupture leads to further atherothrombotic narrowing, while acute hypertension results in the spontaneous rupture of fragile collateral vessels [2, 7]. Further, another theory suggests that emboli to marginally perfused vessels can cause necrosis and rupture [3, 16]. In case 1 in our study, ICA occlusion had an influence on the hemispheric cerebral blood flow of both the occluded and contralateral sides. We speculate that increased contralateral leptomeningeal flow towards the occluded side may have caused cSAH. The establishment of collateral circulation can lead to hemodynamic changes on the contralateral side and leptomeningeal anastomoses on the cerebral surface may have served as a link for this increase in cerebral blood flow [17]. Moreover, occlusion on the ischemic side may have led to the dilatation of the contralateral vasculature as a compensatory mechanism. This dilatation beyond physiological limits increases vessel fragility, and finally causes the ruptures of small vessels. Regarding case 2, the hemodynamic changes due to occlusion of ICA may also have participated in the pathogenesis of cSAH. In addition, we considered that emboli from the heart may have clogged the ipsilateral vessels and caused infarction. Moreover, these emboli may have damaged already highly dilated contralateral vasculature in the arterial border zone, leading to cSAH. Nevertheless, the precise mechanism by which this occurs remains unclear.

Generally, antithrombotic treatment is suggested for patients with infarction. However, the increased risk of cerebral hemorrhage or SAH due to antithrombotic therapy cannot be ignored [18]. There is no consensus on the treatment for acute ischemic stroke co-occurring with cSAH. Few studies suggest that antithrombotic agents are safe for patients with this condition who have large artery stenosis or occlusion [5, 7–9]. Antiplatelet or anticoagulation drugs did not seem to influence the outcome in our two cases. Both patients had a good prognosis which is consistent with the literature [3, 5, 7–9].

Our case reports suggest that contralateral cSAH can occur alongside acute ischemic stroke caused by atherosclerotic ICA occlusion and cardioembolic stroke. cSAH did not lead to poorer outcomes in our patients. Future research should focus on establishing a standard treatment strategy for this condition.

## Data Availability

The datasets generated during the current study are not publicly available for the sake of protecting patient privacy but are available from the corresponding author on reasonable request.

## Abbreviations

CAA: Cerebral amyloid angiopathy
cSAH: Convexal subarachnoid hemorrhage
CT: Computed tomography
ICA: Internal carotid artery
MRI: Magnetic resonance imaging
RCVS: reversible cerebral vasoconstriction syndrome
SAH: subarachnoid hemorrhage

## References

[1] Kumar S, Goddeau RP, Selim MH, Thomas A, Schlaug G, Alhazzani A (2010). Atraumatic convexal subarachnoid hemorrhage: clinical presentation, imaging patterns, and etiologies. Neurology..

[2] Cuvinciuc V, Viguier A, Calviere L, Raposo N, Larrue V, Cognard C (2010). Isolated acute nontraumatic cortical subarachnoid hemorrhage. AJNR Am J Neuroradiol.

[3] Spanou I, Vassilopoulou S, Koroboki E, Tountopoulou A, Velonakis G, Mitsikostas DD (2017). Convexity subarachnoid hemorrhage due to Cardioembolic stroke in a woman with thyrotoxicosis: alpha case report. J Stroke Cerebrovasc Dis.

[4] Usmani N, Ahmad FU, Koch S (2015). Convexity subarachnoid hemorrhage in ischemic stroke. J Neurol Sci.

[5] Lee MH, Kim SU, Lee DH, Kim YI, Cho CB, Yang SH (2016). Evaluation and treatment of the acute cerebral infarction with Convexal subarachnoid hemorrhage. J Cerebrovasc Endovasc Neurosurg.

[6] Chandra RV, Leslie-Mazwi TM, Oh D, Mehta B, Yoo AJ (2011). Extracranial internal carotid artery stenosis as a cause of cortical subarachnoid hemorrhage. AJNR Am J Neuroradiol.

[7] Zhao H, Han J, Lu M, Zhang Y, Fan D (2017). Incidence and possible causes of nontraumatic convexal subarachnoid haemorrhage in Chinese patients: a retrospective review. J Int Med Res.

[8] Fukuma K, Ihara M, Tanaka T, Morita Y, Toyoda K, Nagatsuka K (2015). Intracranial cerebral artery dissection of anterior circulation as a cause of convexity subarachnoid hemorrhage. Cerebrovasc Dis.

[9] Nakajima M, Inatomi Y, Yonehara T, Hirano T, Ando Y (2014). Nontraumatic convexal subarachnoid hemorrhage concomitant with acute ischemic stroke. J Stroke Cerebrovasc Dis.

[10] Introna A, Mezzapesa DM, Petruzzellis M, Savarese M, Chiumarulo L, Zimatore DS, et al. Convexal subarachnoid hemorrhage and acute ischemic stroke: a border zone matter? Neurol Sci. 2019. 10.1007/s10072-019-03868-6 30937557

[11] van Gijn J, Rinkel GJ (2001). Subarachnoid haemorrhage: diagnosis, causes and management. Brain..

[12] Khurram A, Kleinig T, Leyden J (2014). Clinical associations and causes of convexity subarachnoid hemorrhage. Stroke..

[13] Beitzke M, Gattringer T, Enzinger C, Wagner G, Niederkorn K, Fazekas F (2011). Clinical presentation, etiology, and long-term prognosis in patients with nontraumatic convexal subarachnoid hemorrhage. Stroke..

[14] Refai D, Botros JA, Strom RG, Derdeyn CP, Sharma A, Zipfel GJ (2008). Spontaneous isolated convexity subarachnoid hemorrhage: presentation, radiological findings, differential diagnosis, and clinical course. J Neurosurg.

[15] Yger M, Zavanone C, Abdennour L, Koubaa W, Clarencon F, Dupont S (2015). Acute headache at emergency department: reversible cerebral vasoconstriction syndrome complicated by subarachnoid haemorrhage and cerebral infarction. Case Rep Emerg Med.

[16] Kleinig TJ, Kimber TE, Thompson PD (2009). Convexity subarachnoid haemorrhage associated with bilateral internal carotid artery stenoses. J Neurol.

[17] Sadato A, Maeda S, Hayakawa M, Adachi K, Toyama H, Nakahara I (2018). Carotid stenting for unilateral stenosis can increase contralateral hemispheric cerebral blood flow. J Neurointerv Surg.

[18] Garbe E, Kreisel SH, Behr S (2013). Risk of subarachnoid hemorrhage and early case fatality associated with outpatient antithrombotic drug use. Stroke..

